# P-362. Utilization of a Safety-Net Health System to Offer Comprehensive Migrant Health Screening, Identify People Living with HIV (PLWH) and Provide Rapid Access to HIV Care

**DOI:** 10.1093/ofid/ofaf695.580

**Published:** 2026-01-11

**Authors:** Karen Montes, Sharon F Welbel, Kerianne Burke, Jonathan Doyle, Alfonso Trevino, Jaime Martinez, Daniel Vittum, Monica Mercon Almeida

**Affiliations:** Cook County Health, Chicago, Illinois; Rush Presbyterian Hosptial, Skokie, IL; The Ruth M. Rothstein CORE Center, Chicago, Illinois; Cook County Health, Chicago, Illinois; Cook County Health, Chicago, Illinois; Cook County Health, Chicago, Illinois; Cook County Health, Chicago, Illinois; Cook County Health, Chicago, Illinois

## Abstract

**Background:**

Political turmoil, socio-economic instability, and ongoing humanitarian crisis in Latin America after the SARS-CoV-2 pandemic drove mass migration to the United States southern border. Starting in Fall 2022, migrants were sent to northern states, including approximately 51,000 to Chicago. Cook County Health, one of the largest Midwest safety-net health systems, created a New Arrivals Clinic (NAC) that receives migrants within 24 hours of arrival and provides health screening, including HIV/STI (Sexually Transmitted Infection) testing. This report describes how this safety-net health system has addressed migrants’ health needs and identified/connected PLWH to care.

**Methods:**

From September 2022 until January 2025, 30,782 migrants received health screening at the NAC. Among them, 14,394 were males, 9,862 females and 13,014 children. HIV positive migrants were linked to our Spanish speaking HIV clinic for free HIV care, medical visits, ART/STI and chronic diseases medications, transportation for appointments, food assistance, and care coordination to assess barriers for engagement in care.

**Results:**

A total of 224 (0.01%) migrants tested positive for HIV. One hundred sixty-eight (75%) were males, 56 (25%) female, with a median age of 31, including 42 (18.8%) under the age of 25. Ninety-nine (44.2%) were newly diagnosed with HIV. At baseline, 77 (34.4%) had undetectable HIV RNA levels (HIV VL) (< 40 copies/mL), 35 (15.6%) had a CD4+ count < 200 cells/uL. One hundred ninety (84.8%) migrants had a subsequent care visit and VL, with 153 (80.5%) of these patients undetectable at follow up, including 91 (61.9%) of those with detectable VL at baseline. STI screening identified 1,787 patients positive for syphilis, 1,177 (66%) received treatment; and 1134 Gonorrhea/Chlamydia, 869 (77%) received treatment.

**Conclusion:**

Addressing migrants' health and offering HIV/STI testing/treatment is vital to diagnose and link PLWH to care. Our robust safety-net system, capable of rapidly providing resources for migrants to maintain/reach undetectable VL, has the potential to improve not only individuals’ health, but to contain the spread of HIV/STIs in the community. Next steps include expanding HIV clinic sites for treatment/prevention and improving access to care in the to the migrant communities.

**Disclosures:**

All Authors: No reported disclosures

